# Synergistic Treatment of Infected Burn Wound Utilizing Maggot Debridement and Acellular Fish Skin Grafting—A Case Report

**DOI:** 10.1093/jbcr/irae128

**Published:** 2024-07-02

**Authors:** Anton Borger, Lorenz Semmler, Felix Bergmann, Paul Supper, Christine Radtke

**Affiliations:** Department of Plastic, Reconstructive and Aesthetic Surgery, Medical University of Vienna, Vienna, 1090, Austria; Austrian Cluster for Tissue Regeneration, 1200, Vienna, Austria; Department of Plastic, Reconstructive and Aesthetic Surgery, Medical University of Vienna, Vienna, 1090, Austria; Austrian Cluster for Tissue Regeneration, 1200, Vienna, Austria; Department of Plastic, Reconstructive and Aesthetic Surgery, Medical University of Vienna, Vienna, 1090, Austria; Austrian Cluster for Tissue Regeneration, 1200, Vienna, Austria; Department of Plastic, Reconstructive and Aesthetic Surgery, Medical University of Vienna, Vienna, 1090, Austria; Austrian Cluster for Tissue Regeneration, 1200, Vienna, Austria; Department of Plastic, Reconstructive and Aesthetic Surgery, Medical University of Vienna, Vienna, 1090, Austria; Austrian Cluster for Tissue Regeneration, 1200, Vienna, Austria

**Keywords:** chronic wound, blood transfusion refusal, conservative treatment

## Abstract

Here, we report about a patient with a full-thickness burn injury of the left lower extremity with approximately 8% of total BSA affected. Initial therapy consisted of necrosectomy and wound coverage with split-thickness graft. The patient developed a wound infection with *Pseudomonas aeruginosa*, resulting in the failure of the skin graft to achieve complete healing. The case was further complicated by the patient’s concurrent presentation of anemia, characterized by a hematocrit level of 19.8% on 11th day after admission. Additionally, the patient refused acceptance of any blood transfusion, adding a significant layer of complexity to the management strategy. In summary, the patient’s critical state required an immediate intervention. Due to the contraindication for a further surgical debridement and autograft, we changed the treatment strategy to a conservative approach. First, the wound was debrided employing maggot therapy 17 days after admission. Subsequently, free soft tissue coverage was accomplished using decellularized fish skin dressings on 45th day. This approach yielded satisfactory wound closure. Following an approximately 2-month hospitalization period (52nd day after admission), the patient was discharged with a stable wound condition, nearing complete healing.

## BACKGROUND

Burn injuries affect patients of all ages and social classes.^1^ While burns limited to superficial partial thickness typically respond well to conservative therapeutic approaches, deeper partial-thickness burns and those of higher degrees require necrosectomy followed by wound coverage for optimal management.^2^ Established surgical treatments include excision of eschar, extending down to vascularized levels of intact dermis or the epifascial layer, particularly in instances where the dermal vascular plexus is damaged.^3^ Wound coverage is typically performed with autologous skin grafts. Despite its well-established and frequent utilization, the use of autologous skin grafts presents notable drawbacks, including donor site morbidity characterized by a substantial secondary wound site. This secondary site is associated with an elevated risk of complications such as infection, bleeding, and pain, as well as potential outcomes such as scar hypertrophy, depigmentation, or hyperpigmentation. Furthermore, the availability of additional harvest sites is limited especially in patients with major burns.^4^ Recently, innovative alternatives to traditional surgical procedures have emerged. These include allogenic,^5^ or xenologous dressings derived from bovine, porcine or acellularized fish skin.^6,7^ Alternatives to surgical debridement exist in enzymatic products or biodebridement with maggots, offering advantages such as reduced bleeding and need for multiple surgeries and a selective debridement of damaged tissue.^8,9^

## CASE REPORT

**Figure 1.**
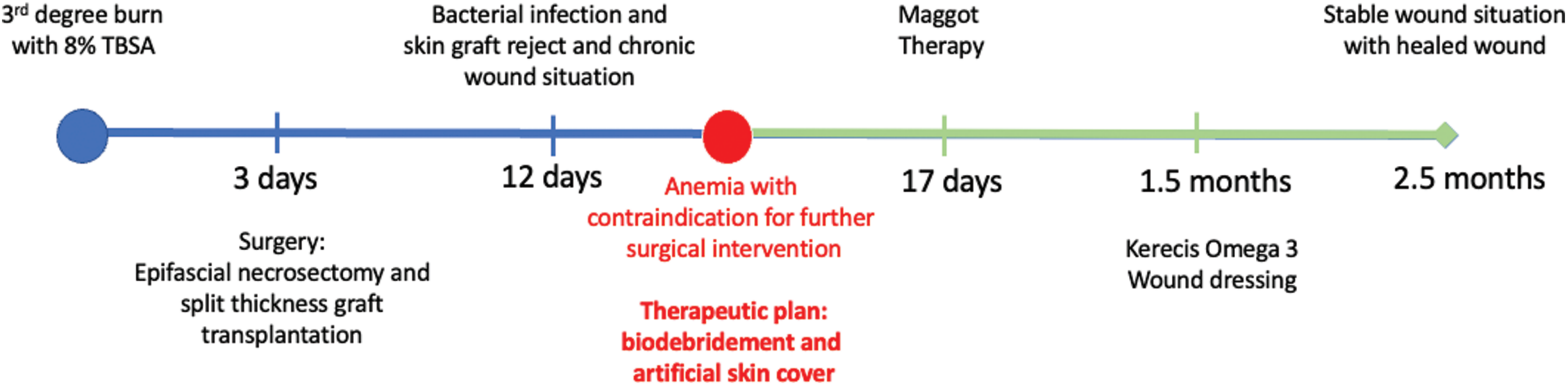
Time Course of Patient’s Healing Process

**Figure 2.**
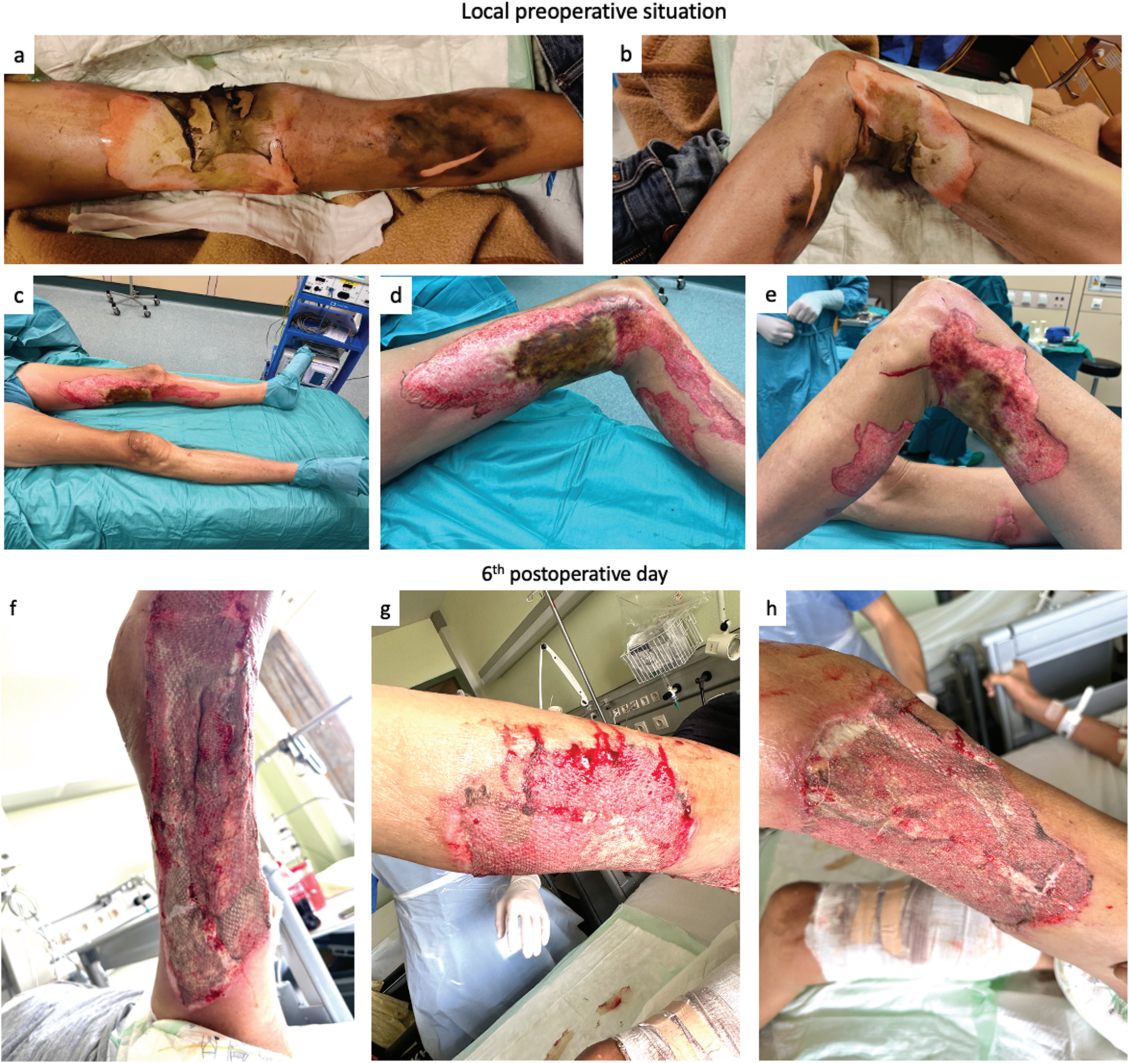
Wound Situation of Burn Injury. (A, B) Local Situation After Admission. (C–E) Injuries After 3 Days and Before Surgical Necrosectomy. (F–H) Wound Situation on 6th Postoperative Day After Skin Graft. (F) Medial and Dorsal Site, (G) Lateral Site of Lower Leg, and (H) Lateral Site of Thigh

We present the case of a 60-year-old male patient who sustained a full-thickness burn injury to his left lower extremity, resulting in a complicated and prolonged course of wound healing and hospitalization (Figure 1). Approximately 8% of the total BSA was affected, with specific areas measuring approximately 60 × 30 cm on the medial side of the thigh and calf, 25 × 30 cm on the lateral side, and 40 × 20 cm on the dorsal side of the thigh (Figure 2A and B). The injury was caused by accidental self-ignition with a lighter while under the influence of alcohol, evidenced by a blood ethanol value of 2.17 ‰. In the patient’s medical history, chronic alcohol abuse and polyneuropathy were noted. Subsequent to first aid performed by paramedics, the patient was transferred to our level I burn injury center.

Following daily dressing changes employing antiseptic fatty gauze (Bactigras, Smith & Nephew, UK), on the third day of admission, the eschar was excised through a tangential and partially epifascial necrosectomy. Subsequently, the wound was covered with meshed split-thickness skin grafts at a ratio of 1:1.5 (Figure 2C and D). The transplanted areas were covered with fatty gauze, a vacuum dressing and splint. The skin donor site from the contralateral thigh was covered with fatty gauze and dry antimicrobial dressings (Kerlix, Medtronic, Ireland). On the fifth day following admission, the patient reported pain in his elbow accompanied by swelling and bruising. An x-ray examination described a preexisting olecranon fracture. The first evaluation of the skin grafts was performed on the 6th postoperative day (POD) (Figure 2F–H).

A decrease in hematocrit level was observed on the fifth day after admission, with values declining from 39.4% (Hb: 14.0 g/dL) preoperatively to 22.6% (Hb: 7.9 g/dL) on the 2nd POD. Subsequently, the hematocrit levels further declined, reaching a value of 19.8% (Hb: 6.6 g/dL) on the 11th day after admission (8th POD) due to the olecranon fixation surgery on the 10th day after admission (7th POD). Consequently, the patient demonstrated symptoms of fatigue and hypotension. However, since the patient adhered to the faith of Jehovah’s Witnesses, any forms of blood transfusions were rejected.

**Figure 3.**
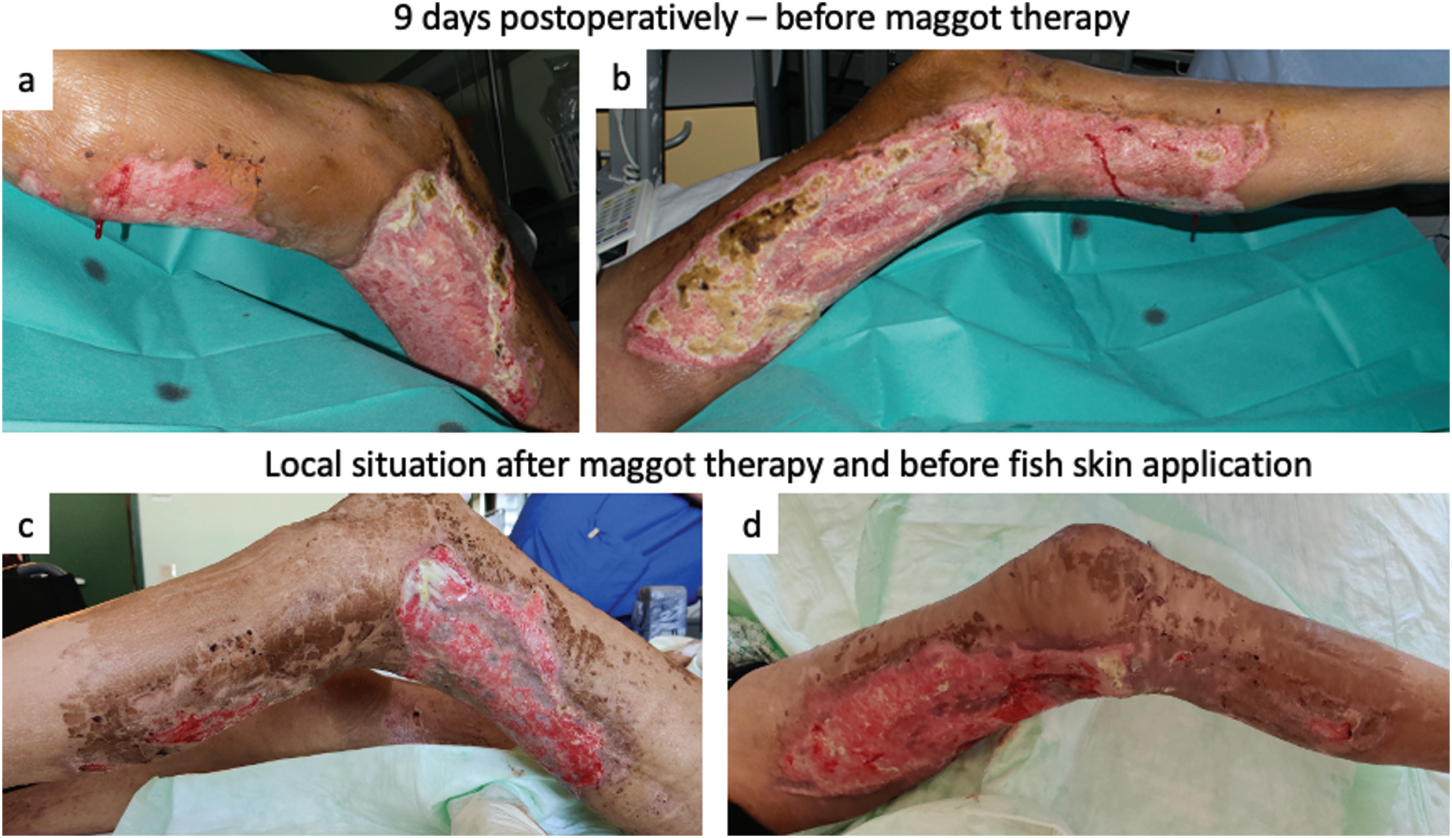
Wound Situation After Split-Thickness Skin Graft and Absent Graft Take. (A, B) Wound Situation 9 Days Postoperatively With a Local Infection and Non-Healed Split-Thickness Skin Graft. (A) Lateral Lower Extremity, (B) Medial Side, and (C, D) Local Situation After Maggot Therapy Treatment

On the 12th day after admission (9th POD), the wound situation at donor and graft sites presented a wound colonization with *Pseudomonas aeruginosa* and *Enterobacter cloacae* (Figure 3A and B). Inflammation parameters in blood showed an increase to 24.34 G/L leukocytes and C-reactive protein levels of 17.74 mg/dL. Consequently, we initiated a regimen of twice-daily irrigation using an antiseptic solution of Betadine and subsequently with hydrophobic bacteria-binding dressings (Cutimed Sorbact Compress, BSN medical, Germany). Furthermore, we intensified the intravenous antibiotic therapy, transitioning from 3 g ampicillin/sulbactam to 4.5 g piperacillin/tazobactam (administered 3 times daily). While the local wound condition at the donor site showed improvement, the graft site exhibited an absence of graft adherence, with significant areas remaining exposed (Figure 3A and B). The soft tissue displayed signs of necrotic damage requiring an immediate surgical debridement. However, due to the persistent low hematocrit levels, further surgical intervention was contraindicated.

A treatment plan involving biodebridement and alternative wound coverage utilizing decellularized fish skin was devised. The patient underwent maggot therapy targeting the medial thigh area (Figure 3B), utilizing *Lucilia sericata* species (Bio Bag, BioMonde, Germany) for a duration of 3 weeks, with weekly changes initiated on the 17th day after admission (14th POD).

**Figure 4.**
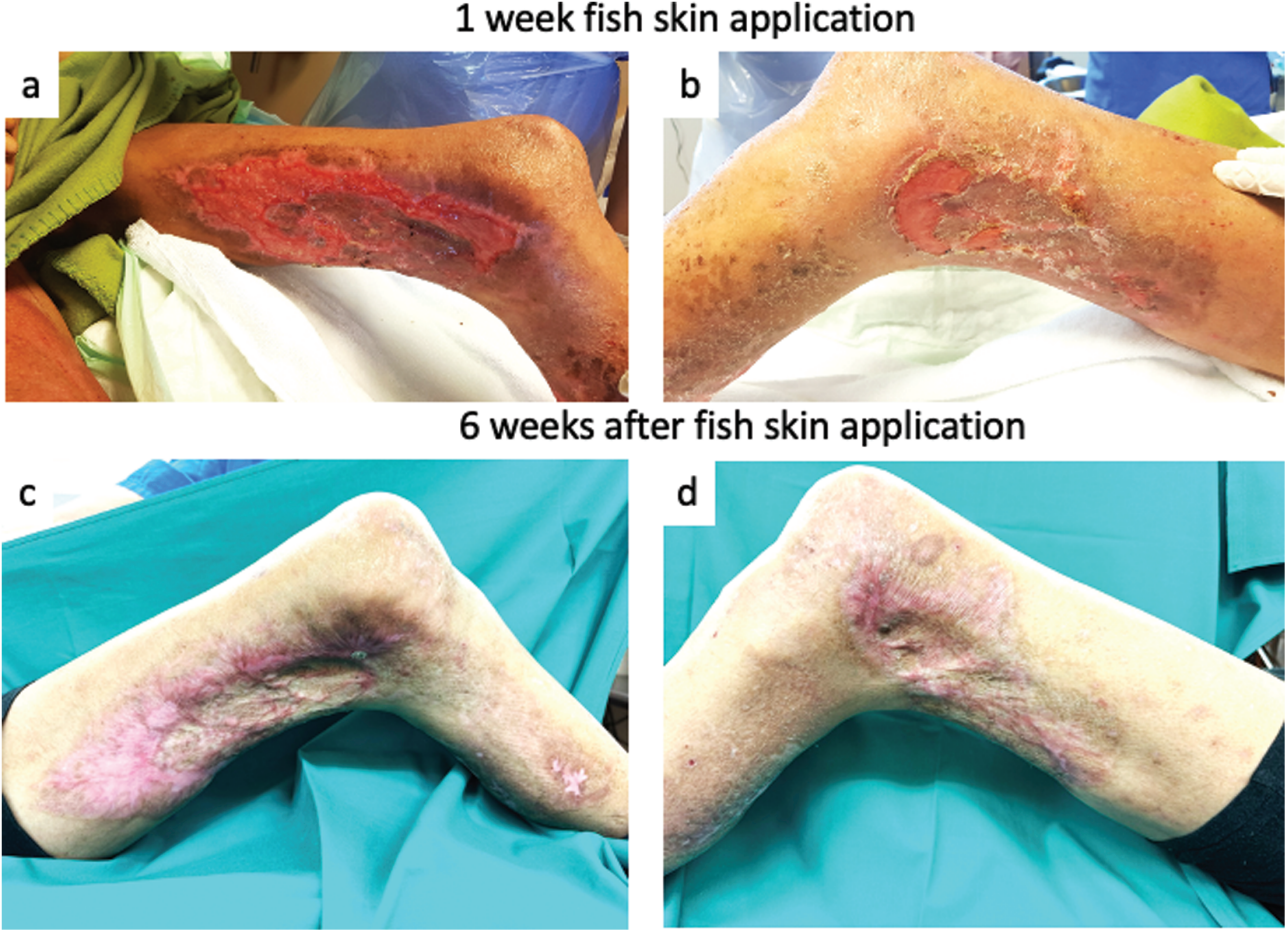
Wound Situation After Fish Skin Dressing Application. (A, B) Local Situation After 1 Week of Kerecis Treatment. (C, D) Situation at the End of Treatment. (A, C) Medial and (B, D) Lateral Site of Thigh

The biodebridement resulted in a wound with an exposed tendon. Thus, treatment with processed fish skin (Kerecis Omega 3 Wound) was initiated in total 6.5 weeks after admission. The fish skin dressing was applied to the wounds (Figure 3C and D) for a duration of 1 week, secured in place using a PICO7 Negative Pressure Wound Therapy System (Smith & Nephew) and the patient was discharged. The wound demonstrated a rapid progression of granulation and reepithelization. The defects on the lateral side, characterized by exposed tendons, exhibited complete granulation and showed a reepithelization to approximately 60%–70% (Figure 4A and B). During outpatient follow-ups, the remaining defects healed following conservative treatment with an absorbing foam dressing (Mepilex, Moelnlycke Health Care GmbH, Austria) after 2 further weeks (Figure 4C and D).

## Discussion

In this article, we present the case of a patient involving biodebridement with maggots and wound coverage using acellular fish skin grafts. In recent literature, acellular fish skin grafts are described as a promising alternative to split-thickness skin grafts for the treatment of dermal defects. Previous clinical studies have demonstrated adequate biocompatibility, and sufficient efficacy in treating burn wounds or chronic ulcers.^10,11^ Moreover, certain studies have indicated that wound healing is superior with acellular fish skin graft compared to those treated with bovine collagen.^7,12^ Further advantages include the absence of a secondary wound site, as previous literature has documented infection rates at donor sites of up to 56%.^4^ Additionally, benefits may arise for patients with concerns regarding the source of wound dressing, such as allogenic, porcine, or bovine origin.

In our case, the patient presented with failed split-thickness skin graft coverage, resulting in exposed soft tissue and tendons. Given the critical anemia observed, further surgical intervention was contraindicated. Furthermore, the patient was susceptible to infections, displaying persistent bacterial colonization of all wound sites. Consequently, we were compelled to transition to a conservative treatment strategy involving maggot therapy and fish skin matrix. The treatment was tolerated well by the patient, who exhibited no adverse events, that is, allergic reactions or zoonotic infections. Our approach resulted in satisfactory wound closure, as evidenced by Figure 3C and D.

In conclusion, our case report highlights the advantages of employing acellular skin grafts and biodebridement, notably in averting the necessity for additional surgical interventions and further wound sites. In summary, conservative approaches involving biodebridement may present a valuable alternative to surgical procedures, particularly for multimorbid patients. However, the extent to which these procedures can entirely replace surgical interventions warrants further investigation through larger clinical trials.

## Data Availability

All data for the study are available upon request to the corresponding author.
